# 不同蛋白A亲和色谱填料的制备工艺比较及性能评价

**DOI:** 10.3724/SP.J.1123.2024.01018

**Published:** 2024-05-08

**Authors:** Linjuan ZHOU, Zhuo WANG, Xingfa REN, Deyun LIU, Lingyi ZHANG, Weibing ZHANG

**Affiliations:** 1.华东理工大学化学与分子工程学院, 上海市功能性材料化学重点实验室, 上海 200237; 1. Shanghai Key Laboratory of Functional Materials Chemistry, School of Chemistry & Molecular Engineering, East China University of Science and Technology, Shanghai 200237, China; 2.月旭科技(上海)股份有限公司, 上海 201613; 2. Welch Technology (Shanghai) Co. Ltd., Shanghai 201613, China

**Keywords:** 亲和色谱, 蛋白A, 琼脂糖, 聚甲基丙烯酸缩水甘油酯, 免疫球蛋白G, 动态载量, affinity chromatography, protein A, agarose, polyglycidyl methacrylate (PGMA), immunoglobulin G (IgG), dynamic binding capacity

## Abstract

蛋白A亲和色谱填料由于能够和免疫球蛋白G(IgG)特异性作用,已广泛应用于临床医学、生物医药领域。采用不同结构和形貌的基质,通过不同的表面修饰方法得到的填料性能差别很大。研究分别以常用的琼脂糖和聚甲基丙烯酸缩水甘油酯(PGMA)小球为基质,通过多功能环氧基定向固定蛋白A制得琼脂糖基质亲和色谱填料(A-S)和PGMA基质亲和色谱填料(P-S)。通过马来酰亚氨基定向固定蛋白A制得琼脂糖基质亲和色谱填料(A-R)和PGMA基质亲和色谱填料(P-R)。通过对蛋白A的键合工艺加以优化,研究了基质种类、基质粒径以及蛋白A含量等条件对材料性能的影响,提高了材料对IgG的吸附性能。结果表明,尽管所制备的4类材料对IgG都有一定的吸附选择性,但P-R的性能明显更加优异,可在较低的蛋白A配基密度下达到较高的动态载量。制备了不同配基密度的亲和色谱填料,其中配基密度为15.71 mg/mL的P-R对牛免疫球蛋白的动态载量为32.23 mg/mL,对人免疫球蛋白的动态载量达到54.41 mg/mL,且在碱处理160个循环后动态载量仍为初始值的94.6%。耐压测试结果表明P-R的机械强度远高于同等条件下制得的琼脂糖基质的亲和材料A-R,当流速到达80 mL/min时,PGMA基质色谱柱的流速与压力仍保持较好的线性关系,P-R的耐压性能好,可以在更高的流速下进行分离纯化。以上结果说明以马来酰亚氨基在PGMA上固定蛋白A的工艺更适宜于蛋白A亲和色谱填料的工程化生产,所发展的方法有望在固定蛋白和免疫吸附材料合成领域发挥更大的作用。

## Abstract (en)

Protein A affinity chromatographic materials are widely used in clinical medicine and biomedicine because of their specific interactions with immunoglobulin G (IgG). Both the characteristics of the matrix, such as its structure and morphology, and the surface modification method contribute to the affinity properties of the packing materials. The specific, orderly, and oriented immobilization of protein A can reduce its steric hindrance with the matrix and preserve its bioactive sites. In this study, four types of affinity chromatographic materials were obtained using agarose and polyglycidyl methacrylate (PGMA) spheres as substrates, and multifunctional epoxy and maleimide groups were used to fix protein A. The effects of the ethylenediamine concentration, reaction pH, buffer concentration, and other conditions on the coupling efficiency of protein A and adsorption performance of IgG were evaluated. Multifunctional epoxy materials were prepared by converting part of the epoxy groups of the agarose and PGMA matrices into amino groups using 0.2 and 1.6 mol/L ethylenediamine, respectively. Protein A was coupled to the multifunctional epoxy materials using 5 mmol/L borate buffer (pH 8) as the reaction solution. When protein A was immobilized on the substrates by maleimide groups, the agarose and PGMA substrates were activated with 25% (v/v) ethylenediamine for 16 h to convert all epoxy groups into amino groups. The maleimide materials were then converted into amino-modified materials by adding 3 mg/mL 3-maleimidobenzoyl-*N*-hydroxysuccinimide ester (MBS) dissolved in dimethyl sulfoxide (DMSO) and then suspended in 5 mmol/L borate buffer (pH 8).

The maleimide groups reacted specifically with the C-terminal of the sulfhydryl group of recombinant protein A to achieve highly selective fixation on both the agarose and PGMA substrates. The adsorption performance of the affinity materials for IgG was improved by optimizing the bonding conditions of protein A, such as the matrix type, matrix particle size, and protein A content, and the adsorption properties of each affinity material for IgG were determined. The column pressure of the protein A affinity materials prepared using agarose or PGMA as the matrix via the maleimide method was subsequently evaluated at different flow rates. The affinity materials prepared with PGMA as the matrix exhibited superior mechanical strength compared with the materials prepared with agarose. Moreover, an excellent linear relationship between the flow rate and column pressure of 80 mL/min was observed for this affinity material. Subsequently, the effect of the particle size of the PGMA matrix on the binding capacity of IgG was investigated. Under the same protein A content, the dynamic binding capacity of the affinity materials on the PGMA matrix was higher when the particle size was 44-88 μm than when other particle sizes were used. The properties of the affinity materials prepared using the multifunctional epoxy and maleimide-modified materials were compared by synthesizing affinity materials with different protein A coupling amounts of 1, 2, 4, 6, 8, and 10 mg/mL. The dynamic and static binding capacities of each material for bovine IgG were then determined. The prepared affinity material was packed into a chromatographic column to purify IgG from bovine colostrum. Although all materials showed specific adsorption selectivity for IgG, the affinity material prepared by immobilizing protein A on the PGMA matrix with maleimide showed significantly better performance and achieved a higher dynamic binding capacity at a lower protein grafting amount. When the protein grafting amount was 15.71 mg/mL, the dynamic binding capacity of bovine IgG was 32.23 mg/mL, and the dynamic binding capacity of human IgG reached 54.41 mg/mL. After 160 cycles of alkali treatment, the dynamic binding capacity of the material reached 94.6% of the initial value, indicating its good stability. The developed method is appropriate for the production of protein A affinity chromatographic materials and shows great potential in the fields of protein immobilization and immunoadsorption material synthesis.

抗体作为识别和分析的重要因子,在生物分子研究和医学诊断、治疗中具有很高的应用价值^[1]^,其中单克隆抗体(mAb)是获批的最大一类生物治疗药物。在过去的30年里,单克隆抗体疗法在医疗保健领域的重要性不断提高^[2]^。目前应用最广泛的抗体纯化方法是基于蛋白A的亲和色谱法^[3⇓-5]^。蛋白A亲和色谱法是一种高选择性的蛋白纯化方法,免疫球蛋白G(IgG)作为主要的血清蛋白,在人类多种疾病治疗中具有独特的优势^[6]^。蛋白A与IgG的Fc区之间有着很强的特异性结合作用,因而偶联蛋白A的亲和填料可用于免疫球蛋白及单克隆抗体的分离纯化,通常通过一步纯化即可达到较高的纯度和产量^[7⇓⇓⇓-11]^。

将蛋白A配体高效连接到基质上是制备蛋白A亲和填料并将其用于高效抗体结合和纯化的先决条件^[3]^。传统的在固体基质上固定蛋白的方法大多是基于非特异性吸附,或是依靠蛋白中天然存在的氨基或羧基与基质上的活化基团反应。蛋白A的分子取向往往是随机的、不受控的,这可能会导致蛋白与基质的结合位点不恰当,从而使蛋白活性下降影响亲和填料的结合性能^[12⇓⇓-15]^。蛋白A配体的定向固定化是亲和色谱中提高抗体结合容量的最有效的方法之一^[16]^。定向固定可使蛋白以一种特定有序的方式固定在基质上,减小蛋白与基质间的位阻,使蛋白的生物活性位点得到很好的保留^[13,17,18]^。von Roman等^[19]^报道了在相同的配体浓度下,定向固定比随机固定具有更好的IgG吸附能力。Shi等^[3]^研究发现,与随机固定相比,定向固定提高了50%的抗体吸附能力。

多功能环氧基两步固定蛋白A以及利用马来酰亚胺与巯基的特异性反应固定蛋白A是常见的定向固定方法。Zhang等^[20]^将环氧活化的琼脂糖表面部分环氧基转化为氨基,制得了多功能环氧基材料,材料表面的氨基通过快速的物理吸附使蛋白A靠近基质,随后材料上剩余的环氧基与蛋白A发生共价反应。重组蛋白A的C端含有一个半胱氨酸,马来酰亚胺能与半胱氨酸上的巯基发生Michael加成反应从而使蛋白A在基质上定向固定^[21,22]^。该反应具有点击化学的特征,有很高的选择性,反应快速且条件温和,被广泛用于含有半胱氨酸的蛋白、多肽等生物分子的偶联。

近年来,蛋白定向偶联技术的发展和应用取得了显著进展^[23,24]^,但大多采用琼脂糖基质来固定蛋白,合成的亲和填料表现出低的机械性能,难以满足在高流速下进行分离纯化的要求^[25]^。本文通过多功能环氧基定向固定蛋白A制得琼脂糖基质亲和色谱填料(A-S)和聚甲基丙烯酸缩水甘油酯(PGMA)基质亲和色谱填料(P-S)。通过马来酰亚氨基定向固定蛋白A制得琼脂糖基质亲和色谱填料(A-R)和PGMA基质亲和色谱填料(P-R)。探究了蛋白A加入量等条件对蛋白偶联量及结合IgG性能的影响,并对比了琼脂糖与PGMA基质的耐压性能。此外,用牛初乳对每种亲和填料的纯化效果和亲和性能进行验证,均取得了令人满意的结果。

## 1 实验部分

### 1.1 仪器、试剂与材料

紫外可见分光光度计(UV-1900)购于日本岛津公司;蛋白纯化仪(Unique AutoPure100-L2)购于苏州英赛斯智能科技有限公司;高速冷冻离心机(TGL-20M)上海卢湘仪离心机仪器有限公司;恒温培养摇床(THZ-300)购于上海一恒科技有限公司;酶标仪(K3)购于赛默飞世尔科技(中国)有限公司;凝胶成像仪(GenoSens 2150)上海勤翔科学仪器有限公司。

环氧活化亲和琼脂糖凝胶介质(Epoxy Purose 4 Fast Flow,粒径45~165 μm)购于嘉兴千纯生物有限公司;牛免疫球蛋白(bovine IgG)和人免疫球蛋白(human IgG)购于上海翌圣生物有限公司;重组蛋白A和PGMA(粒径88~154 μm)由月旭(上海)科技有限公司提供;亲和色谱填料(TOSOH TOYOPEARL AF-rProtein A HC-650F)购于泰渡生物科技(苏州)有限公司;二甲基亚砜(DMSO)、乙二胺(EDA,纯度98%)购于上海麦克林生化科技股份有限公司;3-马来酰亚氨基苯甲酸-*N*-琥珀酰亚胺酯(MBS,纯度98%)、三羟甲基氨基甲烷(Tris)、硼酸、硼砂、乙醇胺、半胱氨酸(cystine)、甘氨酸(glycine)、溶菌酶、牛血清白蛋白(BSA,纯度98%)、磷酸氢二钠、磷酸二氢钠、氯化钠、乙醇等均购于上海阿拉丁试剂有限公司;BCA蛋白浓度测定试剂盒P0011购于上海碧云天生物技术有限公司;Millipore超滤管(50000 MWCO)、0.45 μm无菌滤膜均购于美国Sigma Aldrich公司。牛初乳样品购于华东理工大学教育超市。

### 1.2 多功能环氧基固定重组蛋白A亲和填料的制备

参考Zhang等^[20]^的方法并优化,在琼脂糖和PGMA上固定蛋白A(图1a)。

**图1:**
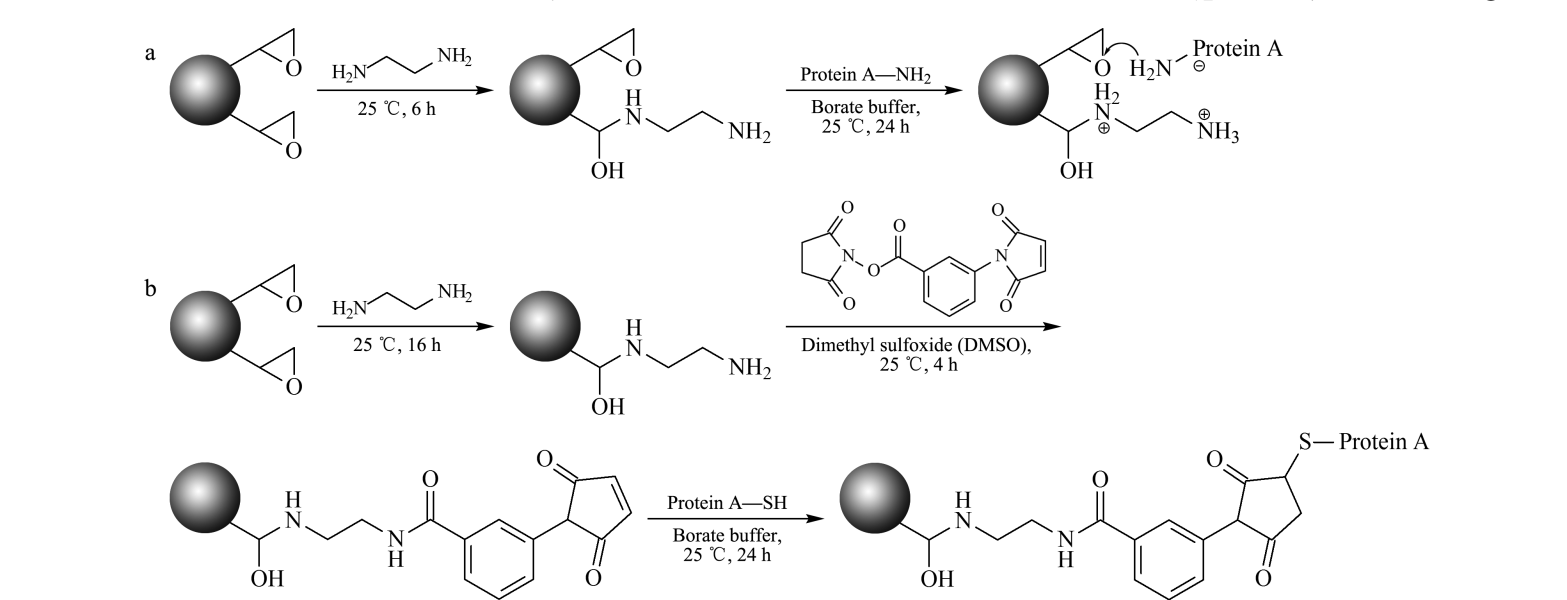
(a)多功能环氧基固定蛋白A和(b)马来酰亚氨基固定蛋白A的合成示意图

取洗净抽干的沉降体积为2.5 mL的琼脂糖/PGMA于15 mL离心管中,加入5 mL 0.2 mol/L(琼脂糖)/1.6 mol/L(PGMA)乙二胺溶液(pH 8.5),于25 ℃反应6 h。反应结束后,用大量超纯水洗涤,直至洗涤液用酚酞指示剂检测不再变红,得到表面部分氨基化的多功能环氧基材料。再加入5 mL用硼酸缓冲液溶解的不同浓度蛋白A,在25 ℃恒温摇床(170 r/min)中反应24 h,反应结束后离心取出上清液。之后加入1.5 mol/L NaCl解吸物理吸附的重组蛋白A,再取出上清液,以BCA试剂盒测定蛋白浓度,计算微球偶联蛋白A的配基密度(*μ*, mg/mL)和偶联效率(*ρ*,%)。最后一步封端,材料用大量超纯水洗净,按材料和溶液体积比为1∶2的比例加入1 mol/L乙醇胺(pH 9)于25 ℃过夜反应,反应结束后用大量超纯水冲洗,制得蛋白A材料,保存于4 ℃、20%乙醇水溶液中。

配基密度计算公式如下:


(1) $$\mu =\frac{m_{1}-C_{1}V_{1}-C_{2}V_{2}}{V_{m}}$$

偶联效率计算公式如下:


(2) $$\rho =\frac{m_{1}-C_{1}V_{1}-C_{2}V_{2}}{m_{1}}\times 100\%$$

其中,*m*_1_为投入蛋白A的质量,mg; *C*_1_、*C*_2_分别是偶联反应上清液和解吸物理吸附蛋白A上清液的质量浓度,g/L; *V*_m_、*V*_1_、*V*_2_分别是基质、偶联反应上清液和解吸物理吸附蛋白A上清液的体积,mL。

### 1.3 马来酰亚氨基固定重组蛋白A亲和填料的制备

参照Duan等^[12]^的方法并优化,在琼脂糖和PGMA上固定蛋白A(图1b)。

取洗净抽干的沉降体积为2.5 mL的琼脂糖/PGMA于15 mL离心管中,加入5 mL 25%(v/v)乙二胺水溶液,于25 ℃反应16 h。反应结束后,用大量超纯水洗涤,直至洗涤液用酚酞指示剂检测不再变红,加入2 mL用DMSO溶解的MBS溶液(3 mg/mL)和3 mL 5 mmol/L 硼酸缓冲液反应4 h,反应结束后加入含有10%(v/v)DMSO的5 mmol/L硼酸缓冲液清洗,再用大量超纯水清洗。

取上述材料加入5 mL 5 mg/mL用硼酸缓冲液溶解的蛋白A溶液,在25 ℃恒温摇床(170 r/min)中反应24 h,反应结束后离心取上清液测蛋白A浓度,计算微球偶联蛋白A的配基密度和偶联效率。最后一步封端,材料用大量超纯水洗净,按材料和溶液体积比为1∶2的比例加入10 mmol/L半胱氨酸于25 ℃过夜反应,反应结束后用大量超纯水冲洗,制得蛋白A材料保存于4 ℃、20%乙醇水溶液中。

### 1.4 吸附性能

#### 1.4.1 静态吸附

取沉降体积为0.25 mL的蛋白A免疫吸附材料于2 mL离心管中,加入1.5 mL 10 mmol/L磷酸盐缓冲液(PBS,含150 mmol/L NaCl, pH 7.4),以1000 r/min平衡1 h,离心弃去PBS后再加入1.5 mL牛免疫球蛋白溶液(6 g/L,以PBS配制),在1000 r/min条件下反应20 h。反应结束后离心弃去上清液,再用PBS清洗材料,最后加入1.5 mL 0.1 mol/L甘氨酸缓冲液(pH 3.0),以1000 r/min转速洗脱20 min。洗脱结束后离心吸取洗脱液并立即用1 mol/L Tris-HCl(pH 8.5)中和防止IgG变性。然后用PBS定容至25 mL,用BCA试剂盒测定蛋白A的质量浓度(*C*, g/L),计算静态载量(SBC)。

静态载量计算公式如下:


(3) $$SBC=\frac{CV_{A}}{V_{B}}$$

其中,*V*_A_为洗脱液定容后的体积,mL; *V*_B_为蛋白A免疫吸附材料的沉降体积,mL。

#### 1.4.2 动态吸附

动态载量(DBC)定义为亲和色谱柱吸附蛋白样品溶液后的流出液吸光度到达测试所用蛋白样品溶液吸光度的10%时蛋白样品溶液的上样量^[26]^。将装有蛋白A亲和填料的1 mL色谱柱接入蛋白纯化仪,用10 mmol/L PBS冲洗色谱柱至少10倍柱体积(*V*_c_)至基线平稳。上样液为10 mmol/L PBS配制的1 g/L牛免疫球蛋白溶液,以0.2 mL/min流速上样,开始上样时系统体积记为*V*_a_,同时在280 nm处检测流出液的吸光度。当流出液吸光度到达牛免疫球蛋白溶液初始吸光度的10%时结束上样,结束上样时系统体积为*V*_b_,再用PBS冲洗至基线平缓,最后用0.1 mol/L甘氨酸洗脱缓冲液以4 mL/min流速洗脱结合的牛免疫球蛋白,收集的洗脱液立即用1 mol/L Tris-HCl(pH 8.5)中和防止IgG变性。实验结束后用PBS冲洗色谱至流出液pH为中性,最后保存于4 ℃、20%乙醇水溶液中防止细菌生长。

动态载量计算公式如下:


(4) $$DBC=\frac{C_{s}\left(V_{b}-V_{a}\right)}{V_{c}}$$

其中,*C*_s_为牛免疫球蛋白溶液的质量浓度,g/L。

### 1.5 蛋白A亲和填料纯化牛初乳中IgG

取3 g牛初乳粉溶于10 mmol/L PBS中,涡旋振荡混匀至充分溶解,定容至30 mL。先用6 mol/L盐酸调节pH至4.5,置于37 ℃水浴中35 min后以9000 r/min离心20 min,弃去沉淀以去除脂肪及酪蛋白,保留上清液。再将上清液用10 mol/L NaOH调节pH至7.0, 9000 r/min离心20 min,弃去沉淀以去除*β*-乳球蛋白,保留上清液。然后将上述上清液倒入50 kDa超滤管中,8000 r/min离心10 min,收集截留液,去除小分子杂蛋白及脱盐浓缩样品。最后将截留液过0.45 μm滤膜除去微生物杂质,准备上样。

以10 mmol/L PBS平衡色谱柱至基线平稳后手动进样1 mL预处理的牛初乳,收集流穿液和洗脱液。预处理的牛初乳原液、流穿液、洗脱液以及1 g/L的牛免疫球蛋白溶液用12%的十二烷基磺酸钠-聚丙烯酰胺凝胶电泳(SDS-PAGE)分析。

### 1.6 填料耐碱性测试

为对比不同方法固定蛋白A制备的亲和色谱填料的性能差异,对配基密度相近的P-S和P-R以及商品化填料HC-650F进行耐碱性测试。待测填料装柱后先用PBS平衡至基线稳定,再用0.5 mol/L NaOH以0.2 mL/min流速清洗15 min为1个循环,每20个循环后用PBS冲洗柱子至中性再以牛免疫球蛋白溶液测动态载量。

### 1.7 填料耐压测试

为对比不同基质的亲和色谱填料的耐压性能,分别对相同条件下制备的A-R和P-R进行耐压测试。将两种填料分别装入体积为1 mL的色谱柱,将色谱柱连入蛋白纯化仪,再以超纯水为流动相,初始流速为1 mL/min,记录不同流速对应的压力值。

## 2 结果与讨论

### 2.1 多功能环氧基固定重组蛋白A

如图1a所示,以环氧活化琼脂糖和PGMA聚合物为基质,重组蛋白A为配体,采用两步法固定蛋白。首先将基质上的部分环氧基转化成氨基,氨基首先通过离子交换物理吸附蛋白A,随后蛋白A上的伯氨基与基质上剩余的环氧基发生反应,最后用乙醇胺将未反应的环氧基封尾制得亲和材料。蛋白A的N端以及侧链中含有多个伯氨基,因此多功能环氧基材料可通过多点共价偶联实现蛋白A的固定,具有一定的定向能力。

#### 2.1.1 多功能环氧基固定重组蛋白A中蛋白含量的影响

配基密度直接影响亲和填料的结合容量,采用不同蛋白含量的蛋白A溶液(1、2、4、6、8、10 mg/mL),固定基质与蛋白A溶液体积比为1∶2,制备了一系列不同蛋白A配基密度的亲和填料,研究了不同蛋白A含量对琼脂糖基质制备的亲和填料(A-S1~A-S6)和PGMA基质制备的亲和填料(P-S1~P-S6)偶联蛋白A效率和结合IgG性能的影响,结果见表1和图2。

**表1:** 多功能环氧基固定法在不同蛋白含量下的偶联效率

Adsorbent	Protein A content/(mg/mL)	Protein A density/(mg/mL)	Coupling efficiency/%
A-S1	1	2.00	100
A-S2	2	4.00	100
A-S3	4	8.00	100
A-S4	6	11.92	99.32
A-S5	8	15.34	95.91
A-S6	10	18.09	90.46
P-S1	1	2.00	100
P-S2	2	4.00	100
P-S3	4	7.92	99.03
P-S4	6	11.53	97.43
P-S5	8	15.40	96.40
P-S6	10	18.40	92.00

A: agarose; P: polyglycidyl methacrylate (PGMA); S: multifunctional epoxide fixation method.

**图2:**
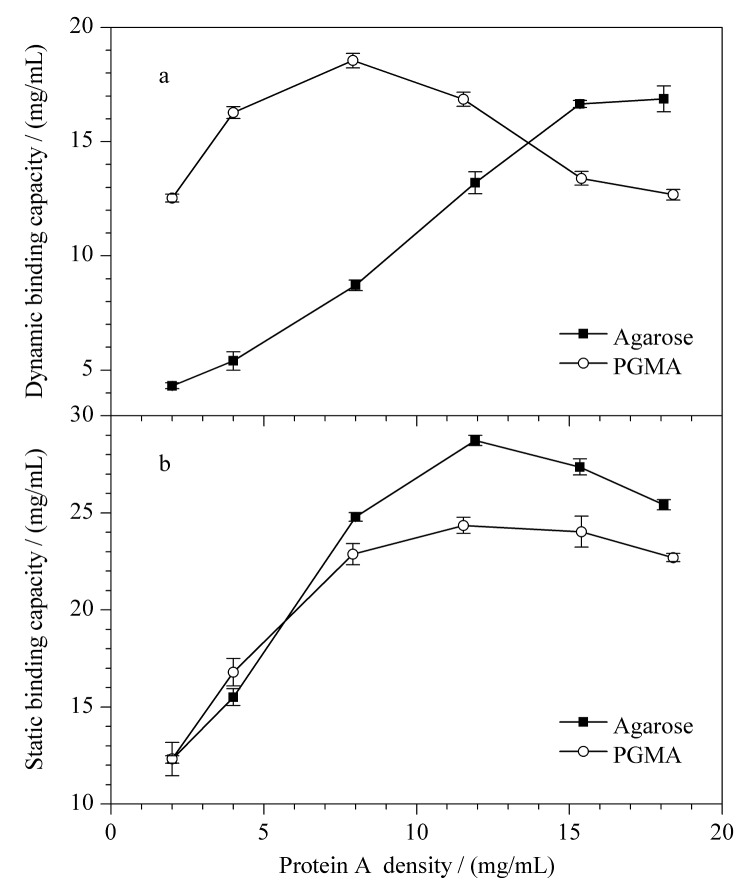
多功能环氧基固定法中蛋白A密度对亲和填料结合牛免疫球蛋白(a)动态载量与(b)静态载量的影响(*n*=3)

从表1可见,无论是琼脂糖还是PGMA基质,偶联蛋白量随着蛋白加入量增大而增大,蛋白A的偶联率逐渐降低。可能是由于琼脂糖和聚合物基质表面的环氧基偶联位点有限,随着蛋白A加入量的增加,大量的蛋白A与环氧基位点发生偶联导致空间位阻增大,蛋白A偶联率降低,这与Haginaka等^[27]^的研究一致。Huang等^[6]^和Katoh等^[28]^在SiO_2_表面偶联蛋白A时也发现了类似的现象。

图2a是不同配基密度对牛免疫球蛋白动态载量的影响。根据图2a,对于琼脂糖基质的蛋白A亲和填料,在蛋白A配基密度为2~20 mg/mL时,随着配基密度的增高,吸附量也随之增大,当蛋白A配基密度达到15.34 mg/mL时,牛免疫球蛋白动态载量达到16.52 mg/mL。对于PGMA基质的蛋白A亲和填料,在配基密度为2~20 mg/mL时,随着配基密度的增高,吸附量先增大后减小最后趋于稳定,当重组蛋白A配基密度为7.92 mg/mL时,牛免疫球蛋白动态载量达到最高值18.36 mg/mL。由于蛋白A配基密度的增加,IgG与蛋白A结合的可能性增加,动态载量随之提高,当蛋白A配基密度进一步增加时可能会产生空间位阻,一定程度上导致了IgG难以进入介质内部并与蛋白A接触,从而导致载量的降低。

图2b是不同配基密度对牛免疫球蛋白的静态载量的影响。根据图2b,两种不同基质的亲和填料在蛋白A配基密度为2~20 mg/mL时静态载量变化趋势基本一致,都是随着蛋白A配基密度的增加,牛免疫球蛋白的载量先增大后趋于稳定。且两种不同基质蛋白A亲和填料的静态载量普遍高于动态载量,这可能是因为静态测试时填料与IgG结合的时间更长,反应更加充分。

不同基质亲和填料的动态载量随蛋白A配基密度变化的差异说明基质对亲和材料的吸附性能有着至关重要的影响,这可能是由于琼脂糖基质与PGMA基质上的环氧基含量不同,且偶联蛋白A所处微观化学环境也存在显著差异。当蛋白A配基密度到达临界值时,由于空间位阻的存在,蛋白A的配基密度增加并不能显著提高亲和填料的吸附性能,反而会增加成本。这与Yang等^[15]^的实验结果基本一致,当蛋白配基密度超过临界密度20 mg/g时,由于严重的空间位阻效应,配体的可利用性迅速下降。

#### 2.1.2 其他条件的影响

乙二胺浓度、偶联蛋白A时的pH和缓冲液的浓度都会影响蛋白A的偶联效率(见附图S1~S3,详见 https://www.chrom-China.com)。研究发现,在两种基质上偶联蛋白最佳的反应液是5 mmol/L硼酸盐缓冲液(pH=8)。对于琼脂糖基质,最佳的乙二胺浓度是0.2 mol/L;对于PGMA基质,最佳的乙二胺浓度是1.6 mol/L。这一差异可能是由于两种基质表面上的环氧基含量不同,而氨基与环氧基在一定的比例下才能达到最佳偶联蛋白效率。

### 2.2 马来酰亚氨基固定重组蛋白A

如图1b所示,以环氧活化琼脂糖和PGMA聚合物为基质,首先将基质表面的环氧基全部转化为氨基,然后加入MBS反应得到马来酰亚氨基填料。利用重组蛋白A的C端半胱氨酸中巯基与马来酰亚氨基特异性反应的特性可定向固定重组蛋白A,最后用半胱氨酸将未反应的马来酰亚氨基封尾。

#### 2.2.1 马来酰亚氨基固定重组蛋白A中蛋白含量的影响

不同蛋白A含量对琼脂糖基质制备的亲和填料(A-R1~A-R6)和PGMA基质制备的亲和填料(P-R1~P-R6)偶联效率和吸附性能的影响见表2和图3。

**表2:** 马来酰亚氨基固定法在不同蛋白含量下的偶联效率

Adsorbent	Protein A content/(mg/mL)	Protein A density/(mg/mL)	Coupling efficiency/%
A-R1	1	1.94	96.95
A-R2	2	3.90	97.52
A-R3	4	7.79	97.41
A-R4	6	11.73	97.76
A-R5	8	15.48	97.49
A-R6	10	18.63	93.15
P-R1	1	1.92	95.93
P-R2	2	3.95	98.65
P-R3	4	7.81	97.70
P-R4	6	10.55	86.19
P-R5	8	13.07	81.69
P-R6	10	15.71	78.84

R: maleimide fixation method.

**图3:**
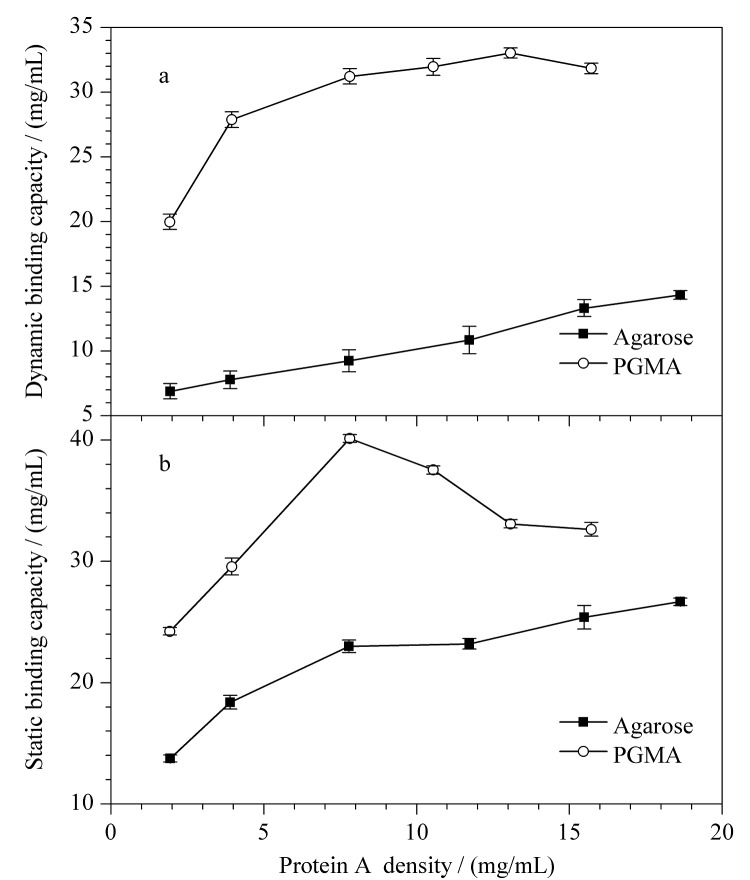
马来酰亚氨基固定法中蛋白A密度对亲和填料结合牛免疫球蛋白(a)动态载量与(b)静态载量的影响(*n*=3)

图3a是马来酰亚氨基固定法中不同配基密度对牛免疫球蛋白的动态载量的影响。根据图3a,对于琼脂糖基质的蛋白A亲和填料,在蛋白A配基密度为2~20 mg/mL时,随着配基密度的增高,载量也随之增大。对于PGMA基质的蛋白A亲和填料,在配基密度为2~20 mg/mL时,随着配基密度的增高,载量先增大后趋于稳定。比较图2a和图3a可以看出,采用PGMA基质通过马来酰亚胺固定蛋白A可以使填料在蛋白A加入量较低时也能达到较高的牛免疫球蛋白动态载量。

图3b是不同配基密度对牛免疫球蛋白的静态载量的影响,两种不同基质蛋白A亲和填料的静态载量均高于动态载量,这与多功能环氧基亲和填料的结果一致。

由图2和图3可以看出,以马来酰亚氨基在PGMA上固定蛋白A制得的亲和填料吸附牛免疫球蛋白溶液的动态载量和静态载量均高于相同条件下在琼脂糖基质上固定蛋白A制得的亲和填料。且以PGMA为基质的马来酰亚氨基免疫吸附材料P-R6,配基密度为15.71 mg/mL,牛免疫球蛋白动态载量为32.23 mg/mL。该动态载量高于相似配基密度下的多功能环氧基亲和填料P-S5(配基密度为15.40 mg/mL,牛免疫球蛋白动态载量为13.05 mg/mL)。这可能是由于重组蛋白A C端的巯基与马来酰亚胺的特异性结合使接枝在PGMA微球表面的蛋白A具有一定的空间构型,从而使蛋白A与IgG的结合位点充分暴露。以人免疫球蛋白为样品测试P-R6,该填料载量达到54.41 mg/mL。由此可见,以马来酰亚氨基固定蛋白A制得的PGMA基质的亲和材料性能更具优势。

#### 2.2.2 基质粒径的影响

基质的粒径对吸附性能也有较大影响。本文研究了两种不同粒径的PGMA基质偶联蛋白的差异,如表3所示,小粒径的PGMA基质结合IgG的能力更好。

**表3:** PGMA基质粒径大小对马来酰亚胺法固定蛋白A的影响

Adsorbent	Particle size/ μm	Protein A content/ (mg/mL)	Protein A density/ (mg/mL)	Coupling efficiency/ %	DBC/ (mg/mL)
P-R-s	44-88	5	9.96	99.61	37.05
P-R-b	88-154	5	9.98	99.87	32.07

### 2.3 重组蛋白A亲和填料的吸附性能

表4比较了不同蛋白A亲和填料的动态载量。亲和填料A-S5和P-R6对人免疫球蛋白的动态载量分别为45.00 mg/mL和54.41 mg/mL,均高于以多功能环氧基固定蛋白制得的亲和填料rSPA-BDA^[29]^、以传统的环氧基固定蛋白制得的亲和填料^[30]^、以半胱氨酸固定蛋白制得的亲和填料Z_Ca_TriCys^[1]^和以羰基咪唑法固定蛋白制得的SiO_2_基质亲和填料^[6]^。且P-R6的动态载量高于以*N*-羟基琥珀酰亚胺酯固定蛋白制得的亲和填料SepFF-oZ4cys^[15]^,说明以马来酰亚氨基在PGMA上固定蛋白A制得的亲和填料性能优异。

**表4:** 本文制备的蛋白A亲和填料与文献中亲和填料的动态载量对比

Affinity packing	Preparation method	IgG DBC/ (mg/mL)
Z_Ca_TriCys^[1]^	via the C-terminal cysteines	15.00 (human)
Protein A-silica^[6]^	via carbonyl imidazole	20.80 (rabbit)
SepFF-oZ4cys^[15]^	via N-hydroxysuccinimide esters	46.70 (human)
rSPA-BDA^[29]^	via the multifunctional epoxide	18.70 (human)
Pharmacia epoxy- activated Sepharose 6B^[30]^	via epoxide	11.00 (human)
P-R6 (this work)	via the maleimide	54.41 (human),
		32.23 (bovine)
A-S5 (this work)	via the multifunctional	45.00 (human),
	epoxide	16.52 (bovine)

取蛋白A配基密度约为15 mg/mL的亲和填料A-S5、P-S5、A-R5、P-R6以及商品亲和填料HC-650F分别装填成1 mL色谱柱后,测得结合牛免疫球蛋白的穿透曲线如图4所示,填充P-R6填料的色谱柱的穿透体积最大,可见以马来酰亚氨基在PGMA上固定蛋白A制得的亲和色谱填料对牛免疫球蛋白的结合性能最好。

**图4:**
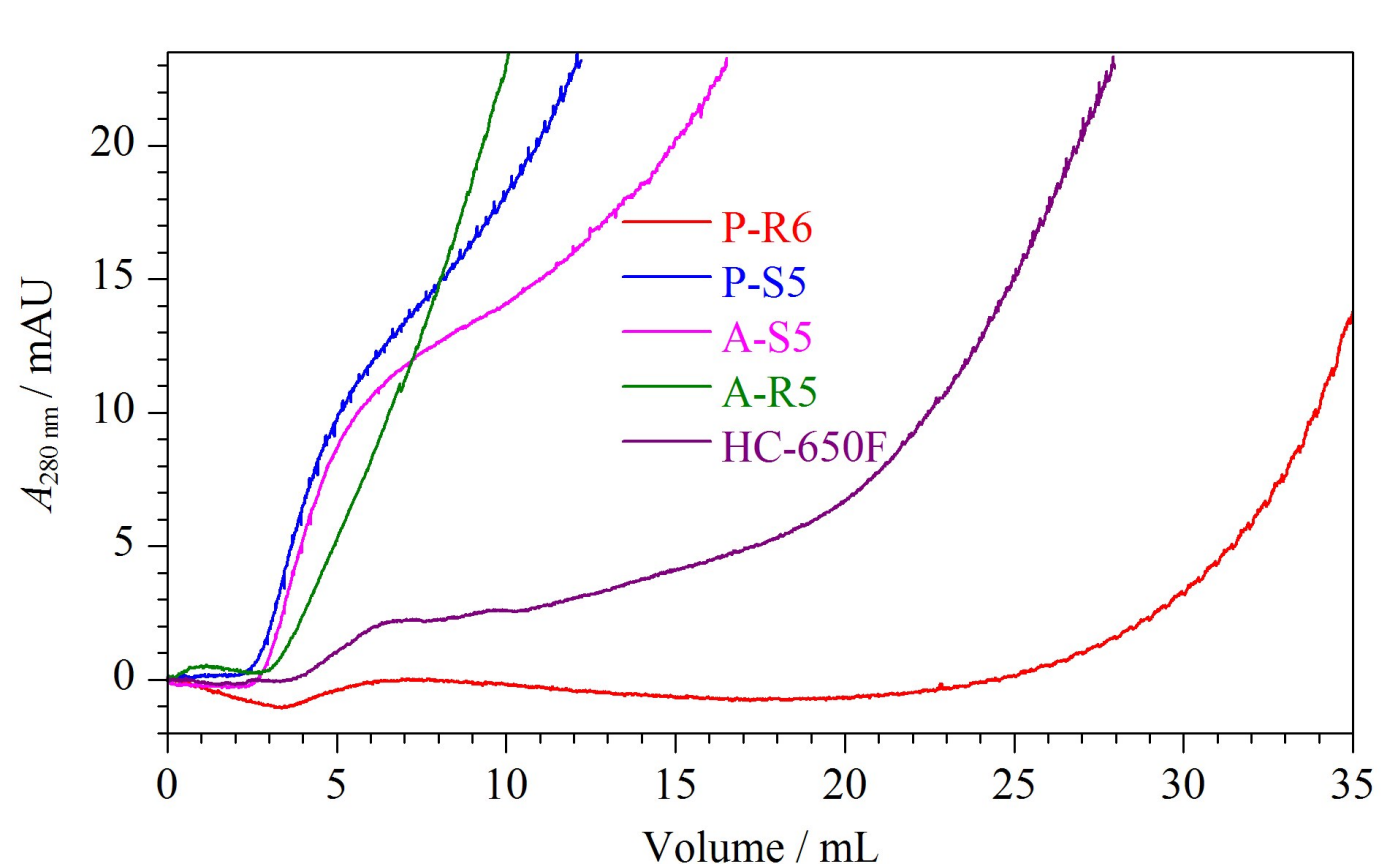
5种亲和填料结合牛免疫球蛋白的穿透曲线

图5是4种亲和填料A-S5、P-S5、A-R5、P-R6纯化的牛初乳中IgG的SDS-PAGE图。牛初乳中含有IgG、牛乳溶菌酶(约18 kDa)、乳铁蛋白(约80 kDa)、乳过氧化物酶(约78 kDa)、牛血清白蛋白(约66 kDa)等多种蛋白。IgG分子由两条重链(55 kDa)和两条轻链(25 kDa)构成。从图5可见,预处理后的牛初乳中蛋白条带复杂。将该牛初乳用上述4种亲和填料纯化后,各洗脱液泳道仅在55 kDa和26 kDa处出现IgG的特征条带,可知各洗脱液中的主要成分是IgG,证明了这4种蛋白A亲和填料对牛初乳中的IgG的特异性吸附。各洗脱液经凝胶成像仪条带分析,纯度分别为91.2%(A-S5)、91.8%(P-S5)、92.8%(A-R5)、94.9%(P-R6)。其中,A-R5与P-R6这两种材料纯化牛初乳中IgG的洗脱液纯度高于A-S5与P-S5。由此可见,以马来酰亚氨基定向固定蛋白A制得的亲和填料在实际应用中效果更好,不同于多功能环氧基材料通过多点共价反应固定,重组蛋白A只有一个巯基可与马来酰亚氨基反应,蛋白A以一种特定有序的方式固定在基质上,减小了蛋白与基质间的位阻,定向效果更佳。

**图5:**
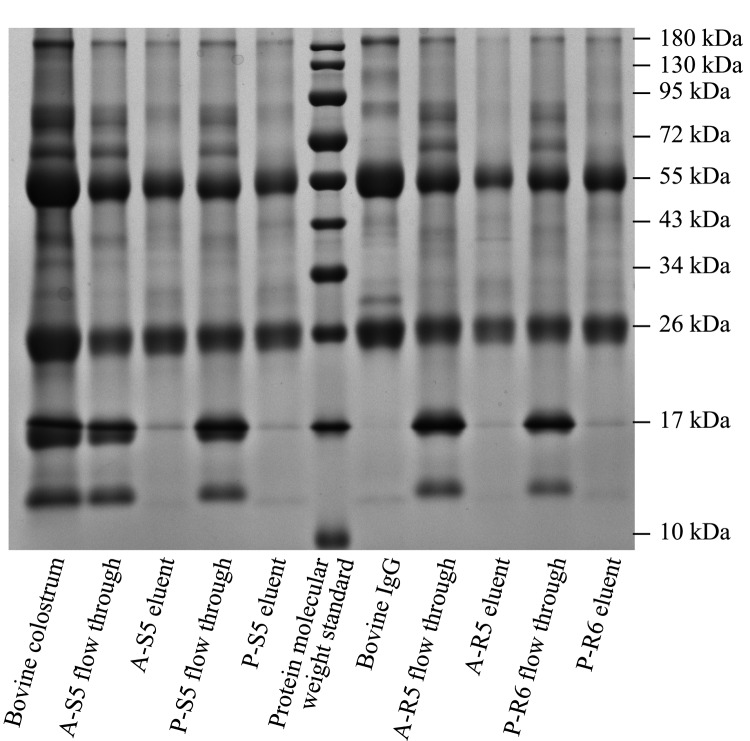
亲和填料纯化牛初乳中IgG的SDS-PAGE图

### 2.4 重组蛋白A亲和填料的耐碱性能

随着亲和填料使用次数的增加,污染物在色谱柱上的积累也在不断增加,定期的原位清洗能有效防止污染物的累积。耐碱性的蛋白A填料可以承受NaOH的反复原位清洗循环,延长纯化周期,同时保持高结合载量^[31]^。如图6所示,用0.5 mol/L NaOH处理160个循环后,再用牛免疫球蛋白测试动态载量,P-R6和P-S5的动态载量分别下降到最初的94.6%和75.9%,商品化HC-650F填料下降到最初动态载量的74.5%。结果表明,以马来酰亚氨基在PGMA上固定蛋白A制得的亲和色谱填料的耐碱性能更优异。

**图6:**
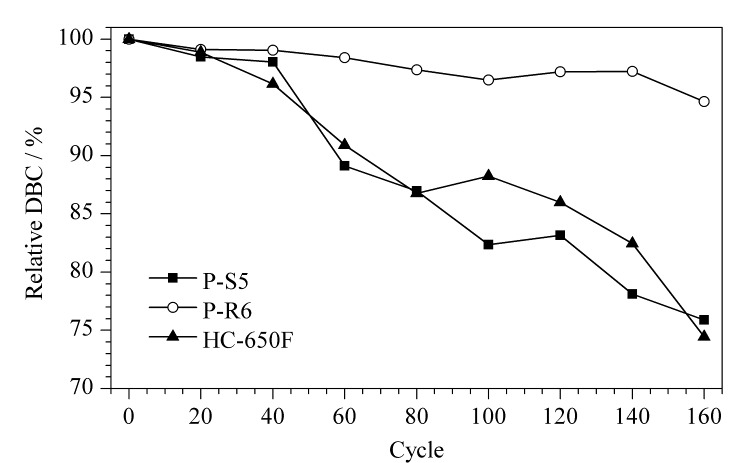
聚合物基质亲和填料的耐碱性

### 2.5 重组蛋白A亲和填料的耐压测试

为对比P-R6和A-R6的耐压性能,在不同流速下测定填料的背压,其结果图7所示。P-R6在流速1~80 mL/min时,其压力≤1.67 MPa。同时,压力可以随着流速的增加保持线性增长。当流速到达80 mL/min时,PGMA基质色谱柱的流速与压力仍保持较好的线性关系,证明其具有良好的机械强度。而A-R6在流速为34 mL/min时,填料已被破坏。由此可见,PGMA基质亲和填料的耐压性能远高于琼脂糖基质,可以在更高的流速下进行分离纯化。

**图7:**
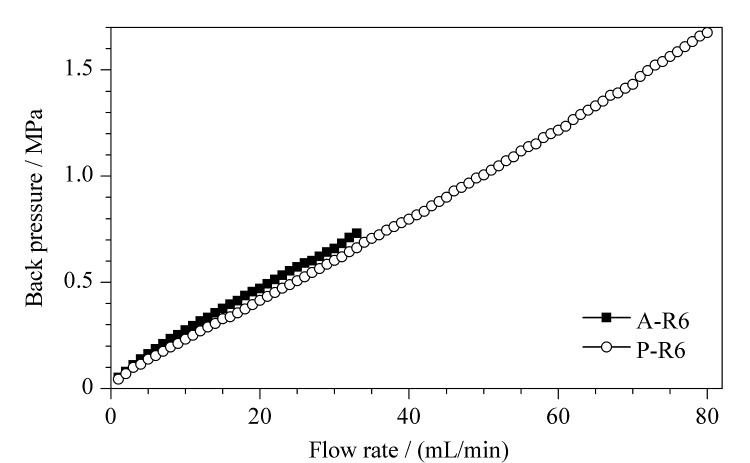
不同基质亲和填料流速与压力的关系

## 3 结论

以琼脂糖和PGMA聚合物为基质通过多功能环氧基和马来酰亚胺定向固定蛋白A制得4类亲和填料,均显示出良好的IgG吸附性能。与多功能环氧基固定蛋白A相比,马来酰亚氨基固定蛋白A的方法定向效果更佳,蛋白A以特定有序的方式固定在基质上,制得的亲和填料对IgG的吸附能力更强。与琼脂糖相比,PGMA的机械强度更大,以PGMA为基质制备的亲和填料更耐压,可在高流速下工作。故以马来酰亚氨基定向固定蛋白A得到的PGMA基质亲和填料有着良好的应用前景,适用于蛋白A亲和色谱填料的工程化生产,所发展的方法有望在固定蛋白和免疫吸附材料合成领域发挥更大的作用。
